# Study on Mg/Al Weld Seam Based on Zn–Mg–Al Ternary Alloy

**DOI:** 10.3390/ma7021173

**Published:** 2014-02-13

**Authors:** Liming Liu, Fei Liu, Meili Zhu

**Affiliations:** Key Laboratory of Liaoning Advanced Welding and Joining Technology, School of Materials Science and Engineering, Dalian University of Technology, Dalian 116024, Liaoning, China; E-Mails: liufei33733@163.com (F.L.); zhuml@dlut.edu.cn (M.Z.)

**Keywords:** Mg/Al welding, multicomponent alloy, intermetallics, microstructure evolution

## Abstract

Based on the idea of alloying welding seams, a series of Zn–*x*Al filler metals was calculated and designed for joining Mg/Al dissimilar metals by gas tungsten arc (GTA) welding. An infrared thermography system was used to measure the temperature of the welding pool during the welding process to investigate the solidification process. It was found that the mechanical properties of the welded joints were improved with the increasing of the Al content in the Zn–*x*Al filler metals, and when Zn–30Al was used as the filler metal, the ultimate tensile strength could reach a maximum of 120 MPa. The reason for the average tensile strength of the joint increasing was that the weak zone of the joint using Zn–30Al filler metal was generated primarily by α-Al instead of MgZn_2._ When Zn–40Al was used as the filler metal, a new transition zone, about 20 μm-wide, appeared in the edge of the fusion zone near the Mg base metal. Due to the transition zones consisting of MgZn_2_- and Al-based solid solution, the mechanical property of the joints was deteriorated.

## 1. Introduction

It has been well known that Al alloys have attractive mechanical and metallurgical properties, including high strength and excellent corrosion resistance, and now, they are widely used for structural components in many applications, such as aerospace, automobiles and the electronics industry [1,2]. Due to Mg alloys’ unique properties, such as a lower weight ratio and electromagnetic shielding capability [3,4], they will have great potential uses in the manufacturing industry. In addition to that, with growing economic and environmental needs, Mg alloys have become the favorite choices in the automobile field. Thus, if Mg alloys were welded to Al alloys to form a compound structure, not only the flexibility and availability of components would be improved substantially, but also the weight and the cost of components would be reduced dramatically [5–7].

At present, welding techniques, such as laser welding [8], soldering [9], vacuum diffusion bonding [10], explosive welding [11] and friction stir welding (FSW) [12–14], are proposed to weld Mg alloys and Al alloys. No matter what kind of techniques are used, if the brittle and hard intermetallics, such as Al_3_Mg_2_ and Al_12_Mg_17_, formed in the Mg/Al weld seam, the performances of welded joints could be deteriorated seriously. Therefore, in order to further improve the tensile strength of Mg/Al joints, it is necessary to reduce the quantity or to change the distribution of Mg–Al intermetallic compounds (IMCs) in the Mg/Al joint.

Previous research has indicated that the formation of Mg–Al intermetallic compounds (IMCs) could be avoided by filling Zn metal in the weld seam in the gas tungsten arc (GTA) welding of Mg and Al alloys [15]. With this method, the weld seams were mainly composed of MgZn_2_, Zn-based solid solution and Al-based solid solution. The continuous layer of MgZn_2_ IMCs locating in the alloying seams was separated by the mixture of the Zn-based solid solution and Al-based solid solution (MZAS). The MZAS with excellent plasticity has been reported by Straumal *et al.* [16], owing to the fact that the Al–Zn and Al–Mg systems are the basis of multicomponent alloys, which present a high strain-rate superplasticity, and having observed the wetting of grain boundaries for these systems, it is suggested that grain boundary pre-melting or pre-wetting is responsible for the high strain-rate superplasticity. This explained why the tensile strength of the Mg/Al joint with Zn filler metal was increased compared with the Mg/Al direct fusion joint. However, the MZAS content in the fusion zone (FZ) near the Mg base metal is less than that in the center of the weld seam and near the Al base metal; in other words, there is the presence of excessive MgZn_2_ in the FZ near Mg base metals, and the MZAS is unable to separate the MgZn_2_ layer effectively. Hence, the FZ near the Mg base metal becomes the weak zone of the joint [17].

In the present work, by estimating the melting quantity of Mg base metal and Al base metal and the distribution ratios of the molten base metal in different locations of FZ, a series of Zn–*x*Al (*x* is the weight percentage) filler metals is designed to accurately modulate the microstructure and composition of the alloyed welding seam, aiming to increase the amount of Al-based solid solution in the weak area of the FZ. An infrared thermography (IRT) system was used to measure the temperature of the welding pool to investigate the solidification process, especially the solidification speed of the welding pool. Additionally, the influences of the Al element on the microstructure of FZ, especially near the Mg base metal, are investigated. Furthermore, the mechanical properties and the fracture mechanisms of the joints will be discussed.

## 2. Experimental Section

### 2.1. Experimental Materials and Instruments

**Figure 1.**
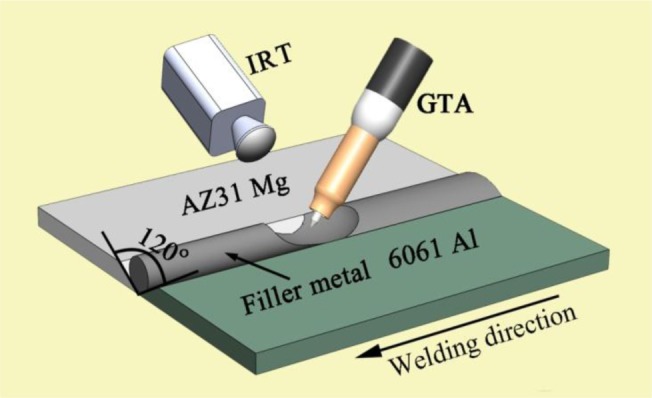
Schematic illustrations of gas tungsten arc (GTA) butt welding. IRT, infrared thermography.

The base metals employed in this investigation were aluminum alloy 6061 sheet and magnesium alloy AZ31B sheet; their nominal compositions are shown in Table 1. The dimensions of the two sheets were 100 mm × 50 mm × 2 mm. Zn–*x*Al alloys were processed into rod-like shapes with a diameter of 3.5 mm as the filler metals. Prior to welding, a “V”-shaped groove of 120 degrees was created, then ground and degreased by an abrasive and acetone, respectively. During the welding, the filler metals were placed in the groove. The IRT system was used for measuring the temperature field by the sampling portion of the emitted energy within a wavelength band of 8–14 μm; each infrared image consists of 240 × 320 discrete temperature sensors. The schematic illustration of the welding process is shown in Figure 1. The GTA parameters used in the experiment were an AC current of 120 A with a welding speed of 450 mm/min. In order to keep the shape of the welding pool constant, the tungsten electrode tip should be ground before every welding.

**Figure 2.**
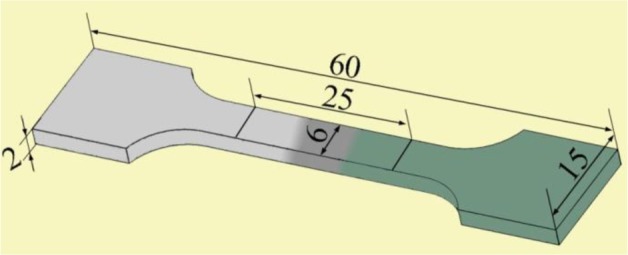
Sketch of butt welding tensile test specimens (mm).

The cross-sections of the welded joints, perpendicular to the welding direction, were prepared in accordance with the metallographic method and etched by 3% HNO_3_ alcohol solution. The structure of the cross-sections and the fracture surfaces of the welded joints were observed under metalloscope and scanning electron microscope (SEM). Element distributions in the welding seam were evaluated with the electron probe micro-analyzer (EPMA)-1600. The phases in the welding seam were analyzed with X-ray diffraction (XRD). According to ASTM: E-8/E8M-11 sub-size specifications [18], the sub-size of the tensile test specimens were manufactured perpendicular to the welded seam with gauge lengths of 25 mm and a width of 6 mm, as shown in Figure 2. The tensile testing was conducted at room temperature under a travel speed of 2 mm/min by a Css-2205 tensile machine. The value of tensile strength is calculated from the average tensile strength of three or more specimens.

An IRT system was used to measure the temperature of the welding pool during welding to investigate the solidification process, especially the solidification speed of the welding pool. The expression of IRT temperature can be calculated using the following equation [19]:

$$T={\left(P_{\left(T\right)}/(\varepsilon \sigma )\right)}^{1/4}$$ (1)

where σ is the Stefan–Boltzmann constant, *P*_(*T*)_ is the radiation intensity dependent on the temperature of the object and ε is the emissivity of a real object. It can be seen from the equation that a traditional IRT system is suitable only for the temperature measurement of single state of phase (solid, liquid or gas). The emissivity of the welding pool changes significantly, with the welding pool transforming from one state to another during the welding process. In the case of Zn–30Al, the emissivity of Zn–30Al filler metal calibrated by the thermocouple is 0.45 under the liquid state and is 0.91 under the solid state. When an IRT system is in use, the emissivity of the welding pool is set to 0.45 for the entire time during the welding process. Assuming the real temperature of the welding pool is *T*_r_ on the moment just after solidification, *T*_r_ can be expressed as $T_{\text{r}}={\left(P_{\left(T\right)}/(0.91\sigma )\right)}^{1/4}$. Additionally, when the welding pool temperature is measured by an IRT system, *T*_m_ is represented as $T_{\text{m}}={\left(P_{\left(T\right)}/(0.45\sigma )\right)}^{1/4}$. It is obvious that the temperature measured by an IRT system (*T*_m_) is higher than the real temperature (*T*_r_) on the moment just after solidification. For this reason, the cooling curve of the welding pool measured by the IRT system does not monotonically decrease during the solidification, which can be used to judge when the solidification of the welding pool begins [19]. In other words, when the temperature curve of solidification appears to be at the turning point, the welding pool is considered to be beginning to solidify. The solidification time of the welding pool can be estimated from the welding speed, which can provide us an auxiliary analysis for the solidification process of the welding pool.

### 2.2. The Design of Zn–xAl Filler Metals

The size of the welding pool in different weld seams is constant when using GTA welding at the same welding parameters [20,21]. In order to control the composition of the Mg/Al welding seam, it is necessary to estimate the melting quantity of the Mg base metal and Al base metal and the distribution ratio of these molten base metals in different locations of the fusion zone. It has been known that the FZ of Mg/Al weld seam is mainly composed of Mg base metal, Al base metal and filler metal. When pure Zn is used as the filler metal to weld Mg alloy and Al alloy together, all the Al and Mg in the weld seam comes from the molten base metal; thus, the dilution of the base metal can be calculated. In this present work, pure Zn filler metal is used to join Mg base metal with Al base metal first, and the distribution ratios of molten base metals in different locations of the fusion zone are calculated. Then, pure Zn is replaced by Zn–*x*Al to accurately modulate the composition of the Mg/Al alloyed welding seam.

**Figure 3.**
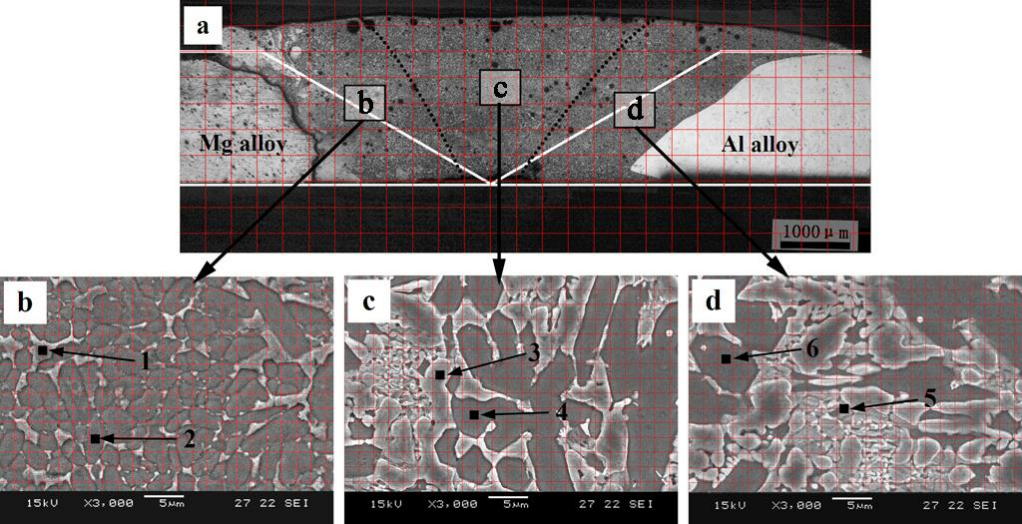
Microstructures of welded joint filling with pure Zn filler metal. (**a**) The macrostructure of the joint; (**b**), (**c**) and (**d**) the magnified images of (**b**), (**c**) and (**d**) in (**a**), respectively.

Figure 3a shows the macrostructure of the welded joint formed between Mg and Al base metal by GTA weld filling with pure Zn filler metal. The white line in Figure 3a is the contour line of the Mg and Al base metal prior to welding. Due to the nonuniform distribution of the molten Mg base metal and Al base metal in different locations of FZ, the area of FZ is equally divided into three parts by dotted black lines in Figure 3a: near the Mg base metal (Area b), in the middle of FZ (Area c) and near the Al base metal (Area d). Figure 3b,c,d are the magnified images of the areas in b, c and d in Figure 3a, respectively. It can be seen form Figure 3b,c,d that the FZ is mainly composed of white phases and grey phases. The area percentages of white phases and grey phases in Areas b, c and d are estimated by the grid method, as shown in Table 2. The compositions of white phases and grey phases are evaluated by EPMA, and the results show that the compositions of the phase with the same color change a little in different locations of the same zone. For this reason, only the compositions of two areas with different colors are shown in each zone. The results are shown in Table 2.

To simplify the calculation, Mg and Al base metals are considered to be pure Mg and pure Al. Based on the data in Table 2, the Mg content and the Al content at different locations of the fusion zone can be calculated by the area percentage and composition of white and grey phases when the pure Zn is used as the filler metal.

**Figure 4.**
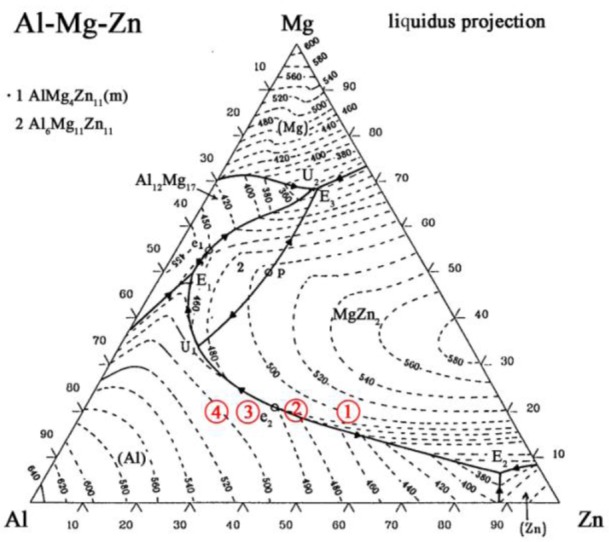
Mg–Al–Zn ternary phase diagram.

In order to increase the content of the Al-based solid solution in Area b, to improve the tensile strength of the Mg/Al joints, pure Zn is replaced by a Zn–*x*Al filler metal to make the contents of Al in the filler metal gradually increase. In the Zn–*x*Al filler metal, *x* is set to 10, 20, 30 and 40 to make the composition of Area b across to line U_1_E_2_ of the Mg–Al–Zn ternary phase diagram shown in Figure 4 to precipitate Al-based solid solution primarily [22]. When Zn–10Al, Zn–20Al, Zn–30Al and Zn–40Al are used as the filler metal, the composition of the weak zone are calculated and shown in Table 3.

The calculation results of the points are stretched to areas to eliminate errors coming from the Mg and Al base metals being considered as pure Mg and Al. The locations of Area b in the Mg–Al–Zn ternary phase diagram using the Zn–*x*Al filler metal can be found according to the proportion of Mg, Al and Zn. As shown by the red circles in Figure 4, Areas 1, 2, 3 and 4 are the compositions of Area b, while using Zn–10Al, Zn–20Al, Zn–30Al and Zn–40Al as the filler metals, respectively. It can be seen that the compositions of Area 3 and 4 are at the left side of line U_1_E_2_, which is to say the Al-based solid solution precipitates first during the welding process; so, it is very promising that the content of Al-based solid solution in Area b can be increased.

## 3. Results and Discussion

### 3.1. Analysis of the Weld Pool Solidification

**Figure 5.**
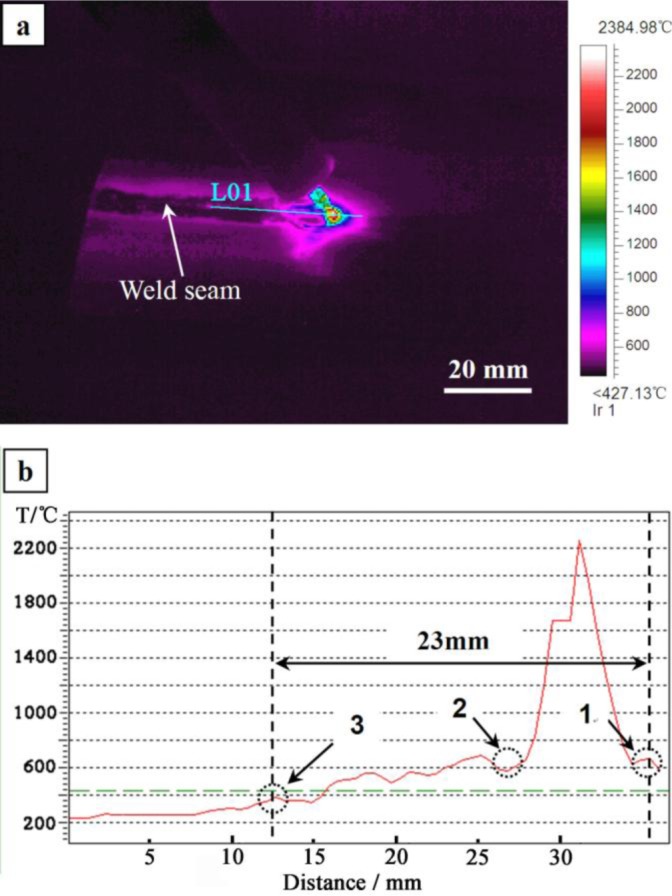
IR image at the welding process: (**b**) the temperature curve of Line 01 (L01) in (**a**).

Zn–30Al filler metal is used as an example to estimate the solidification time of the welding pool. Figure 5a is the infrared thermography captured during welding. Figure 5b is the temperature curve of Line 01 (L01) in Figure 5a, which is generated by the IRT system automatically. It can be seen from Figure 5b that the segment from Point 1 to Point 3 in the temperature curve is the process of melting and solidifying of the welding pool during the welding. According to the deduction of Section 2.1, turning Points 1 and 2 in the temperature curve are the starting points of melting and solidification, respectively. Since the temperature of the welding pool is monotonically decreasing from the center to the edge of welding pool in the real situation, whereas the temperature begins to increase from turning Points 1 and 2 on the temperature curve, so Points 1 and 2 can be considered as the starting points of the melting and solidification, respectively. The temperature curve is monotonically decreasing from Point 3, indicating that the solidification of the welding pool has finished.

The distance from Point 1 to Point 3 is 23 mm, as shown in Figure 5a. The welding speed is 450 mm·min^−1^ (7.5 mm·s^−1^), and the duration of the welding pool is about 3.1 seconds (23 mm/7.5 mm·s^−1^) from calculation using the Zn–30Al filler metal. The solidification of the welding pool is an unsteady process, due to the fast cooling rate of the welding pool. Therefore, the microstructures generated in the weld seam during solidification require examination and not just reference to the Mg–Al–Zn ternary phase diagram.

### 3.2. Microstructure of the Welded Joints

**Figure 6.**
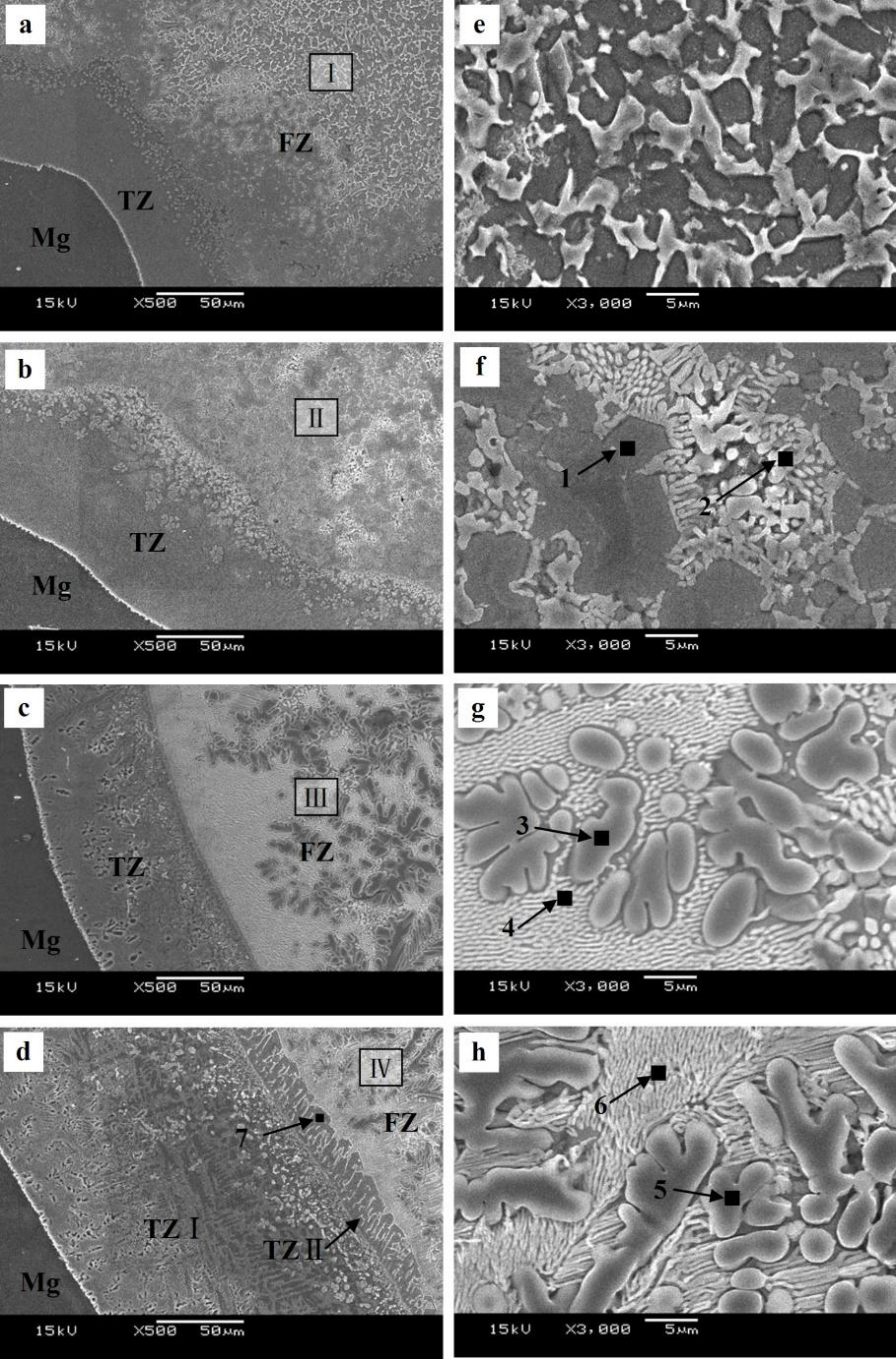
Microstructures of the welded joint in Area I: (**a**, **b**, **c** and **d**) the microstructure of Area I using Zn–10Al, Zn–20Al, Zn–30Al and Zn–40Al as the filler metals, respectively; (**e**, **f**, **g** and **h**) the magnifications of I, II, II and IV from (**a**), (**b**), (**c**) and (**d**), respectively.

**Figure 7.**
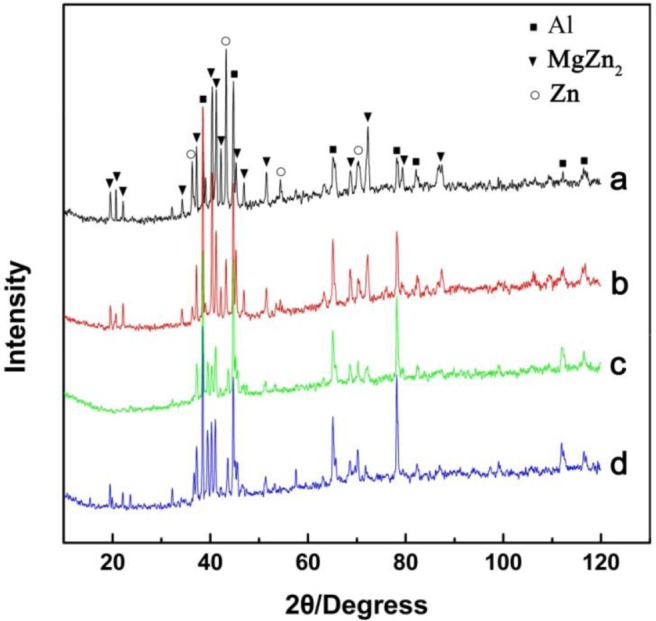
X-ray diffraction patterns of the fusion zone: (**a; b; c** and **d**) the diffraction patterns of the fusion zone using Zn–10Al, Zn–20Al, Zn–30Al and Zn–40Al as the filler metals, respectively.

The microstructure evolutions of the weak area near the Mg base metal in the fusion zone using Zn–*x*Al filler metals were observed by SEM, and the results are shown in Figure 6. Figure 6a is the microstructure of the FZ using Zn–10Al as the filler metal. Figure 6e is the magnified image of Area I in Figure 6a. Similar to the microstructure using pure Zn as the filler metal shown in Figure 3b, Area I is composed of white phases and grey phases. According to the X-ray diffraction patterns of Figure 7a and previous research [15], it is deduced that the grey grains are composed of MgZn_2_ and a little bit of Zn-based solid solution, while the white phases are a mixture of Zn-based solid solution and Al-based solid solution (MZAS). However, the amount of white phases in Area I using Zn–10Al as the filler metal are more than those of using pure Zn as the filler metal, resulting from the increased amounts of Al-based solid solution.

Figure 6b is the microstructure of the area near the Mg base metal in the FZ using Zn–20Al filler metal. Figure 6f is the magnified image of Area II in Figure 6b. Owing to the increased Al content in the filler metal, the amount of grey phases decrease in Area II, as shown in Figure 6f compared with Figure 6e.

The composition of the weak area in the location of the Mg–Al–Zn ternary phase diagram is shown by Area 2 in Figure 4, when the Zn–20Al filler metal is used (see Section 2.2). According to the vertical cross-section of the Mg–Al–Zn ternary phase diagram, it can be inferred that the equilibrium reactions of the weak area in the order of their occurrence during the solidification process are [22]:

**Table:** 

L→MgZn_2_	490°C
L→(Al)+MgZn_2_	475°C
L→(Al)+MgZn_2_+(Zn)	460°C
L→(Al)+MgZn_2_+Mg_2_Zn_11_+(Zn)	340°C

However, because the solidification time of the welding pool is only 3.1 s, the products in the FZ may be different from that of equilibrium solidification. For this reason, the compositions of the white phases and the grey phases in Areas II–IV were evaluated by EPMA, and the results are shown in Table 4. The phases in the FZ with different filler metals were analyzed with XRD, as shown in Figure 7, and Figure 7a–d are the diffraction patterns of FZ using Zn–10Al, Zn–20Al, Zn–30Al and Zn–40Al as the filler metals, respectively. According to the EPMA and XRD results, the firstly precipitation grey phases in Figure 6f are mainly composed of MgZn_2_ and the mixture of Al-solid solution and Zn-solid solution.

The rod-like eutectic phases (white phases) located in the grey phase gaps are the mixture of Al-based solid solution and Zn-based solid solution, as pointed out by Arrow 2 in Figure 6f, and the white phases are increased compared with that of the Zn–10Al used, as shown in Figure 6e.

Figure 6c,g is the microstructures of the area near the Mg base metal in the FZ using the Zn–30Al filler metal, and Figure 6g is the magnified images of Area III in Figure 6c. It can be seen from Figure 6c that the microstructure of the weak area is different from those of Figure 6a,b. When the Zn–30Al filler metal is used, the composition of the weak area of FZ in the location of the Mg–Al–Zn ternary phase diagram is shown by Area 3 in Figure 4 (see Section 2.2), and the equilibrium solidification of the welding pool near the Mg base metal is given by:

**Table:** 

L→(Al)	495°C
L→(Al)+MgZn_2_	480°C
L→(Al)+MgZn_2_+(Zn)	337°C

It can be seen from Figure 6g that the welding pool near the Mg base metal precipitates primarily α-Al instead of MgZn_2_ using Zn–30Al filler metal, as expected. In Table 4, the compositions of α-Al are 73.86 at% Al and 23.04 at% Zn, 3.11 at% Mg, indicating that the solid solubility of Zn in Al is about 23.04%, which is higher than the highest solid solubility of Zn in Al (16.5%) at equilibrium [23]. Thus, supersaturated α-Al formed under welding conditions. The phases, shown by Arrow 4 in Figure 6g, are Al–Zn hypoeutectic and a small amount of MgZn_2_ compound.

Figure 6d is the microstructure of the area near Mg base metal in the FZ using Zn–40Al as the filler metal. It can be seen that there are two transition zones (TZs) (TZ I and TZ II) between the Mg base metal and FZ, which is one more than the other filler metals used. The width of TZ I is 150 μm, and TZ II is 20 μm. Figure 6h is the magnified image of the Area IV in Figure 6d. The phases in Area IV are composed of α-Al and the mixture of Al–Zn hypoeutectic and MgZn_2_, as shown in Area 5 and 6 in Figure 6h. According to the vertical cross-section of the Mg–Al–Zn ternary phase diagram, it is the same as the equilibrium solidification process of the welding pool near the Mg base metal using Zn–40Al as the Zn–30Al filler metal.

Comparing with Figure 6g,h, the α-Al and Al–Zn hypoeutectics are coarser than that of the Zn–30Al used. This is because the initial solidification temperature of α-Al increased with the improvement of Al content in Zn–40Al filler metal, and then, the solidification time of the welding pool extended to 3.5 s, as measured [22]. Since the atoms of the Mg base metal have more time to diffuse into the fusion zone, the TZ I is wider than that of other filler metals, and the reason for the formation of TZ I is similar to that of other filler metals used. In addition, the growth orientation of the grain in TZ II has a certain direction, which is perpendicular to the edge of TZ I, and the reason for the forming of TZ II is that the grains in TZ II have more time to grow, due to the solidification time of welding pool being extended.

The compositions of TZ II were evaluated by EPMA, as shown by Arrow 7 in Figure 6d. The analysis results indicated that TZ II is composed of MgZn_2_ and Al-based solid solution. The content of MgZn_2_ is so high that these hard intermetallic compounds act preferentially as the source of microcracks in the mechanical property tests. As a result, the welded joints with the Zn–40Al filler metal fractured in TZ II.

### 3.3. Tensile Strengths and Fractures of the Welded Joints

**Figure 8.**
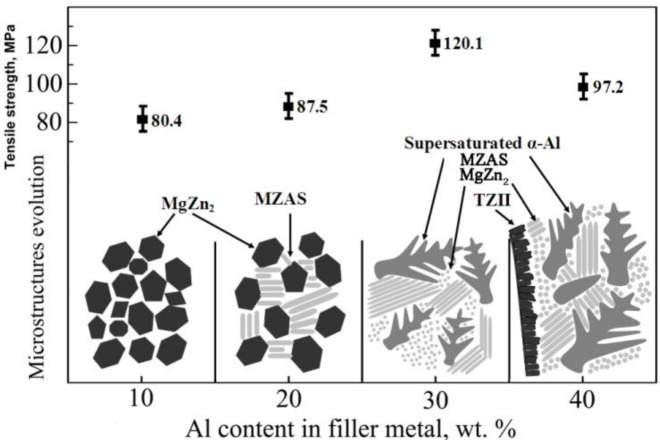
Tensile strength and microstructure evolution of joints with different Al contents in filler metal.

Tensile tests were carried out to measure the tensile strengths of Mg/Al joints with the Zn–*x*Al filler metal. The tensile strengths of materials are determined by their microstructures, so the microstructure evolution of weak zones of joints with different Al content in the filler metal are investigated; and the tensile strengths and microstructure evolution schematic diagrams are shown in Figure 8.

The average tensile strengths of the joints with Zn–10Al and Zn–20Al filler metals are 80.4 and 87.5 MPa, respectively, and the weak zones of the joints are mainly composed of MgZn_2_ and MZAS, according to previous analysis. The tensile strength of the joint is improved with the increasing of the Al content in the filler metal, owing to the MZAS in the joint being increased. The reason is that the alloys, whose compositions are approximate to those of MZAS, have excellent plasticity [16]. When the sample bears the tension stress, the MZAS can eliminate the stress concentration of the crack tip and, hence, hinder crack propagation, and then, the tensile strength of the joint is increased. Another reason is that the grain boundaries in the FZ can be either completely or incompletely wetted by the melt. The phenomenon of grain boundary wetting and wetting phase transitions in the FZ can drastically change their properties. The grain boundaries between MgZn_2_ compounds and solid solution in the joints with the Zn–30Al and Zn–40Al filler metals have a good wettability, due to the solidification time of the welding pool being extended [24].

As shown in Figure 8, the weak zone of the joint generates primarily α-Al instead of MgZn_2_ with the Zn–30Al filler metal, and the average tensile strength increases to a maximum value of 120.1 MPa. However, the average tensile strength of the joint reduces to 97.2 MPa when the Zn–40Al filler metal is used, resulting from TZ II being mainly composed of the MgZn_2_ compound and leading to the joints being fractured at TZ II, whereas the joints are fractured at the FZ near the Mg base metal with other filler metals.

**Figure 9.**
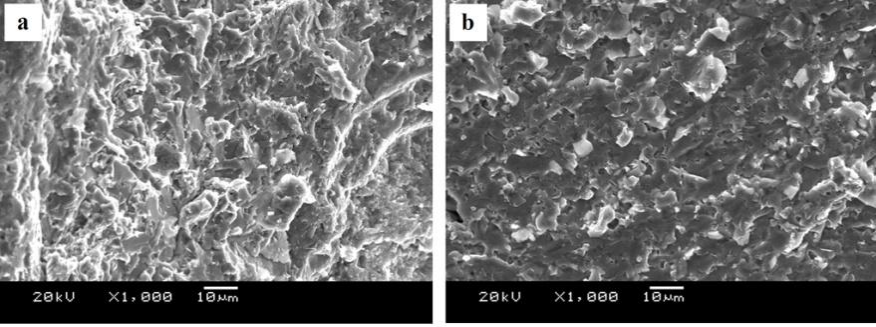

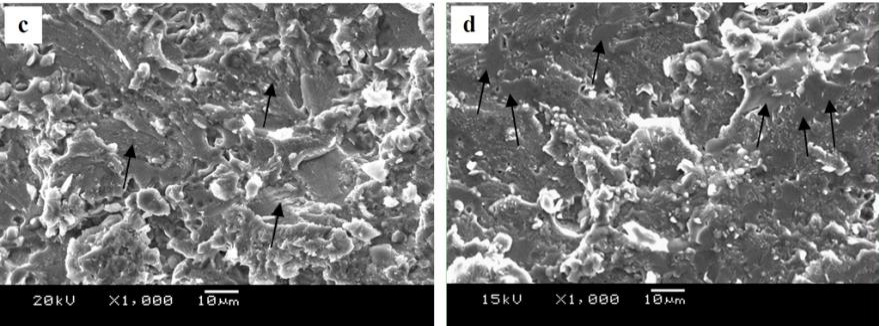
Fracture surfaces of joints with different Al content in the filler metal: (**a**) Zn–10Al; (**b**) Zn–20Al; (**c**) Zn–30Al and (**d**) Zn–40Al.

The fracture surfaces of joints with different filler metal are shown in Figure 9. It can be seen from Figure 9a,b that the fracture surfaces of welded joint with Zn–10Al and Zn–20Al filler metals display a typical brittle fracture feature, indicating that no plastic deformation occurred before fracture. The reason is that the weak area of the joint has plenty of hard and brittle MgZn_2_ compounds and has a little bit of Al-based solid solution and Zn-based solid solution, as shown in Figure 8. Therefore, when the samples bear the tension stress, the solid solution cannot eliminate the stress concentration of the crack tip and, hence, hinder crack propagation; then, this leads to the fracturing of joints, presenting a brittle characteristic.

Figure 9c is the fracture of the joint using Zn–30Al filler metal. It can be seen that the fracture surface has a small amount of cleavage facets (pointed out by the black arrow) and no longer the presence of an absolute brittle fracture feature (a quasi-cleavage feature). When Zn–40Al filler metal is used, a lot of facets on the fracture surface are observed, as pointed out by the black arrow in Figure 9d. Since the joints with the Zn–40Al filler metal fractured at TZ II, where the area is mainly composed of MgZn_2_, it can be deduced that the facets are MgZn_2_ compounds. These MgZn_2_ compounds deteriorate the performances of welded joints seriously and lead to a brittle, fractured surface.

## 4. Conclusions

Based on the idea of alloying a welding seam, the filler metal was designed in advance for joining Mg/Al dissimilar metals by gas tungsten arc welding, and the plates of AZ31B alloy and 6061 alloy were successfully welded using different filler metals. The major conclusions of this study can be summarized as follows:

(1) The composition of the fusion zone can be predicted and controlled by designing different filler metals, and the microstructure and phase of the weak area can be accurately modulated combining the solidification time of the welding pool and the equilibrium phase diagram.
(2) When using Zn–10Al and Zn–20Al as the filler metals, the weak area of FZ precipitated primarily MgZn_2_, and the fracture surface of the joint displayed a brittle fracture. When using the Zn–30Al as the filler metal, the weak area of the joint precipitated primarily α-Al, and the joints presented quasi-cleavage fracture. However, when using the Zn–40Al as the filler metal, the TZ II near the FZ formed, which is mainly composed of the MgZn_2_ compound and the joint fractured at this location.
(3) The tensile strengths of the joints are increased with the increase of Al content in the filler metal. The maximum average strength achieved is 120.1 MPa using Zn–30Al filler metal. However, when using Zn–40Al as the filler metal, the tensile strength of the joint is decreased, due to the formation of TZ II near the FZ.

## Figures and Tables

**Table 1.** Chemical compositions (wt%) of the 6061 Al alloy and AZ31 Mg alloy.

Materials	Al	Mg	Si	Zn	Cu	Mn
6061 Al	remainder	1.00	0.60	–	0.15	0.01
AZ31 Mg	3.00	remainder	0.10	1.00	–	0.20

**Table 2.** The area percentages and compositions of the phases in different locations.

Location in Figure 3a	Phases	Area	Area percentage (%)	Percentage composition (at%)		
				n_Mg_	n_Al_	n_Zn_
(**b**)	white phases	33	10	13.40	43.10	42.50
	grey phases	297	90	40.20	7.50	52.30
(**c**)	white phases	164	50	6.20	7.00	86.80
	grey phases	166	50	34.10	4.80	61.10
(**d**)	white phases	100	30	7.30	26.20	66.50
	grey phases	230	70	31.60	1.20	67.20

**Table 3.** The calculated results.

*x* value		*x* = 10	*x* = 20	*x* = 30	*x* = 40
Filler metal		Zn–10Al	Zn–20Al	Zn–30Al	Zn–40Al
Composition of Area b	Mg	0.199	0.198	0.202	0.203
	Al	0.303	0.402	0.488	0.558
	Zn	0.499	0.399	0.310	0.236
Location in Figure 4		Area 1	Area 2	Area 3	Area 4

**Table 4.** The chemical compositions of different areas.

Serial number	Percentage composition (at%)			Inference composition
	Mg	Al	Zn	
1	17.07	13.11	69.82	(Al), MgZn_2_, (Zn)
2	6.55	33.98	59.47	(Al), Mg_2_Zn_11_, (Zn)
3	3.11	73.86	23.04	α-Al
4	13.30	25.91	60.79	(Al), MgZn_2_, (Zn)
5	3.14	78.62	18.24	α-Al
6	14.27	27.28	58.45	(Al), MgZn_2_, (Zn)
7	23.52	19.92	56.57	(Al), MgZn_2_
